# Lipids and apolipoproteins C-III and E among treatment-naïve and treatment-experienced persons with HIV in Nigeria

**DOI:** 10.4102/ajlm.v12i1.2018

**Published:** 2023-07-17

**Authors:** Mercy N. Okunorobo, Nwakasi K. Nnamah, Ugomma A. Ude, Enyioma A. Ude

**Affiliations:** 1 Department of Medical Laboratory Science, Faculty of Health Sciences and Technology, Nnamdi Azikiwe University, Awka, Nigeria; 2 Department of Chemical Pathology, Faculty of Clinical Medicine, Nnamdi Azikiwe University, Awka, Nigeria; 3 Department of Medical Laboratory Science, Faculty of Health Sciences and Technology, Ebonyi State University, Abakaliki, Nigeria

**Keywords:** dyslipidaemia, apolipoprotein C-III, apolipoprotein E, HIV, antiretroviral therapy, cardiometabolic disorders, lipid profile

## Abstract

**Background:**

Dyslipidaemia is a known cause of cardiovascular mortality. Persons living with HIV are at high risk of developing cardiovascular disease due to lipid metabolism disorders associated with HIV or its therapy.

**Objective:**

This study evaluated concentrations of lipoproteins and apolipoprotein C-III and E, as a way of assessing cardiometabolic risks among HIV patients.

**Methods:**

We enrolled 50 HIV-negative persons and 100 HIV-positive patients, 50 on antiretroviral therapy (ART) and 50 treatment-naïve persons, from the Central Hospital and the Stella Obasanjo Hospital, Benin City, Edo State, Nigeria, between May 2015 and November 2015. Participants with a history of metabolic abnormalities were excluded. Apolipoproteins were assessed by enzyme-linked immunosorbent assay, while lipids were measured by spectrophotometry.

**Results:**

There were significant abnormalities in the lipid profile of patients with HIV. Triglycerides levels of HIV patients (ART-naïve: 1.44 ± 0.65 mmol/L; *p* < 0.001 and ART-experienced: 1.49 ± 0.70 mmol/L; *p* = 0.001) were significantly higher than among controls (0.95 ± 0.54 mmol/L). HIV patients had higher concentrations of apolipoprotein C-III than controls (*p* < 0.001) and higher low-density lipoprotein cholesterol levels (treatment-naïve: 2.83 mmol/L and ART-experienced patients: 3.59 mmol/L) than controls (2.50 mmol/L; *p* = 0.003). Conversely, HIV patients had significantly lowered high-density lipoprotein cholesterol levels compared to controls (*p* < 0.001).

**Conclusion:**

Dyslipidaemia was observed among HIV participants, irrespective of their ART experience. Therefore, it is crucial that the lipids of HIV patients be closely monitored to enable early intervention and decrease cardiovascular death.

**What this study adds:**

This study affirms that dyslipidemia is a complication of HIV or the prolonged use of ART.

## Introduction

HIV is a global pandemic associated with aberrations in the immune system and biochemical parameters, which increases the vulnerability of the infected individual to diseases.^1,2^ According to the Nigeria HIV/AIDS Indicator and impact survey, HIV is highly prevalent among adults in the south-south region of Nigeria.^3^ The use of antiretroviral therapy (ART) in the management of HIV has improved the life expectancy of persons living with HIV^4^ but has also been associated with an increased risk of metabolic abnormalities, including dyslipidaemia and cardiovascular death.^5,6,7^ A higher burden of cardiovascular disease has been reported among HIV patients on highly active antiretroviral therapy treatment compared to highly active antiretroviral therapy-naïve patients.^8^

Dyslipidaemia refers to abnormal plasma lipid concentrations, including elevated plasma total cholesterol, low-density lipoprotein cholesterol (LDL-C), triglycerides, and reduced high-density lipoprotein cholesterol (HDL-C). These could occur as single entities or in combination.^9,10^ Dyslipidaemia is a known predictor of endothelial dysfunction, cardiovascular disease, and atherosclerosis. It is a common non-communicable disease among persons living with HIV. Thirty percent of persons living with HIV have been reported to have dyslipidaemia. The use of ART may contribute to the development of dyslipidaemia in persons living with HIV.^8^ Dyslipidaemia has been reported among both treatment-naïve and ART-experienced HIV-positive patients,^10^ suggesting that lipid abnormalities among HIV patients may be caused by HIV itself and may be worsened by ART.^11^

Changes in lipid synthesis or transportation can cause dyslipidaemia.^12^ Consequently, changes in apolipoproteins, protein components of lipoproteins that bind and transport lipids for metabolism or utilisation, could modulate serum lipid concentrations causing dyslipidaemia. Apolipoprotein E (AE) is essential for the catabolism of remnant (triglyceride-rich) lipoproteins as it is a major transporter of cholesterol and triglyceride-rich lipoproteins.^13^ Apolipoprotein C-III (ACIII) plays a crucial role in the synthesis and catabolism of very low-density lipoproteins.^13^ These proteins have been associated with hypertriglyceridaemia,^14,15^ a known predictor of cardiovascular disease.^16^ Aside from its role in lipid and lipoprotein metabolism, AE is also involved in the immune response by stimulating or inhibiting antigen-T-lymphocyte proliferation.^17^

Studies assessing serum lipid levels of HIV-positive subjects abound. However, there is a paucity of research evaluating apolipoprotein concentrations, despite their importance in lipid concentrations and metabolism. Little is known about the concentration of apolipoproteins and their role in the course of HIV infection. The study aimed to assess the risk of cardiometabolic disorder and determine the correlation between lipids and the studied apolipoproteins of HIV-positive patients.

## Methods

### Ethical considerations

Ethical study approval was sought and obtained from the Hospital Management Board of Edo State, Nigeria (study approval number: CH/A406 VOLIII/177A). The study participants were educated on the study’s details and assured of the confidentiality and anonymity of the information and data obtained during the study. We enrolled only participants who consented in writing. Data that could be traced back to the participants were not collected. All information obtained from the participants was treated with the utmost confidentiality and used strictly for research.

### Study location

The study was carried out at the Heart to Heart Clinic of Central Hospital and Stella Obasanjo Hospital, both in Benin City, Edo State of Nigeria, between May 2015 and November 2015. Both hospitals have major HIV and AIDS centres serving patients from all over the state.

### Study design

This cross-sectional study compared the lipid profile and ACIII and AE levels of HIV-positive and HIV-negative participants. For the case group, 100 participants were HIV-positive and grouped based on their treatment experience. Group one included 50 HIV-positive ART-naïve individuals. In contrast, group two included 50 HIV-positive individuals receiving first-line ART made up of a combination of two nucleotide reverse transcriptase inhibitors (zidovudine or tenofovir and lamivudine) with one non-nucleotide reverse transcriptase inhibitor (efavirenz). All case participants were adults and enrolled for care in the study’s hospitals.

For the control group, 50 HIV-negative adults with no history of organ or metabolic disease were recruited from among staff and patient relatives in the hospitals.

Any individual with a known history of diabetes mellitus or on treatment for dyslipidaemia was excluded from the study. Also excluded were current smokers and excessive alcohol drinkers. Excessive alcohol drinking was defined as a pattern of alcohol use, including binge consumption (four drinks or 70 g [for men] and 56 g [for women] in two hours), frequent or heavy consumption, hazardous use and dependency^18^.

### Sample processing and analytical methods

Five millilitres of blood was obtained after an overnight fast from each of the participants. The blood sample was withdrawn from the antecubital vein using sterile hypodermic syringes into plain containers. All samples were transported from the collection site in the cold chain to the Chemical Pathology Laboratory of a Teaching Hospital in South East Nigeria, where the analysis was done.

The blood was allowed to clot, and the serum separated from the cells by centrifuging at 1500 revolutions per minute for 5 min. The serum was stored at −20 ° C for one week before the samples were analysed.

#### HIV screening

All enrolled participants were tested to confirm their HIV status per the national HIV testing algorithm^19^ using HIV Determine™ (Alere Medical Co. Limited, Tokyo, Japan), Uni-gold Recombigen HIV 1/2 test (Trinity Biotech, Bray, Ireland), and HIV 1/2 STAT-PAK (Chembio Diagnostic Systems, Medford, New York, United States).

#### Anthropometric variables

Anthropometric data of the participants, including height and weight, waist circumference, and systolic and diastolic blood pressure, were measured, and body mass index was computed using the ratio of weight (kg) to height (m^2^).

#### Lipid and apolipoprotein measurement

Lipid profile parameters were estimated using commercially available reagents (Randox Cholesterol, Triglycerides, HDL-Cholesterol) from Randox Laboratories, Crumlin, Ireland. To estimate of total cholesterol and HDL-C, the method of Allan et al.^20^ was employed, while the method of Bucolo and David^21^ was used to estimate triglyceride. Low-density lipoprotein cholesterol was calculated using the Friedewald equation:^22^

$$\text{LDL-C}=\text{TCHOL}–\text{HDL-C}–\frac{\text{TG}}{2.2}$$ [Eqn 1]

In equation 1, LDL-C is low-density lipoprotein, TCHOL is total cholesterol, TG is triglyceride and HDL-C is high-density lipoprotein.

Apolipoprotein C-III and AE were measured by the enzyme-linked immunosorbent assay method using commercially available reagents from ABCAM (Cambridge Biomedical Campus, Cambridge, United Kingdom). Commercially sourced control sera (Randox Laboratories, Crumlin, Ireland) were included in every test, and the routine quality control procedures were maintained. Dyslipidaemia was defined as elevated total cholesterol (≥ 5.0 mmol/L), elevated LDL-C (≥ 3.4 mmol/L), elevated triglyceride (≥ 1.6 mmol/L) or as low HDL-C (≤ 1.04 mmol/ L). Hyperglycaemia was defined as fasting blood glucose ≥ 6.2 mmol/L. Elevated blood pressure was defined as systolic and diastolic pressure greater than 130/85 millimetre of mercury.

### Statistical analysis

Data analysis was conducted using Statistical Package for Social Sciences statistical software version 20 for Windows 7 (IBM Corp., Chicago, Illinois, United States). The results were expressed as mean ± standard deviation (s.d.). Data obtained from this study were analysed using one-way analysis of variance, and post hoc tests were used to compare means. Pearson bivariate correlation was used to assess associations between protein levels and lipid profiles. A *p*-value was considered significant at ≤ 0.05.

## Results

### Characteristics of study population

The mean ages were 39.36 ± 7.02 years for ART-naive, 38.46 ± 7.70 years for ART-experienced and 31.20 ± 7.26 years for HIV-negative participants (Table 1). The mean SBP of the ART-naive participants was 135.52 ± 24.79 millimetres of mercury, 143.26 ± 27.98 millimetres of mercury for ART-experienced participants and 130.72 ± 14.56 millimetres of mercury for the HIV-negative participants. Patients on ART had significantly higher SBP compared to the control group (*p* = 0.023). No significant difference was observed when the SBP of the ART-naïve participants was compared with that of controls (*p* = 0.905) or with the ART-experienced participants (*p* = 0.291).

**TABLE 1:** Socio-clinical characteristics and lipid profile of controls, antiretroviral therapy-naïve and treatment-experienced HIV-positive patients, Benin City, Nigeria between May 2015 and November 2015.

Variables	Healthy HIV-negative controls		Treatment-naïve HIV-positive participants		Treatment-experienced HIV-positive participants		Post hoc test *p*-value			
	Mean	s.d.	Mean	s.d.	Mean	s.d.	Case† vs HC	TN vs TE	TN vs HC	TE vs HC
**Socio-clinical characteristics**										
Age (years)	31.20	7.26	39.36*	7.02	38.46*	7.70	< 0.001	1.000	< 0.001	< 0.001
Systolic blood pressure (mmHg)	130.72	14.56	135.52	24.79	143.26*	27.98	0.026	0.291	0.905	0.023
Diastolic blood pressure (mmHg)	85.50	14.40	91.42	21.85	92.60	17.72	0.117	1.000	0.321	0.161
Weight (kg)	64.86	12.11	65.10	16.18	62.90	12.26	0.677	1.000	1.000	1.000
Height (m)	1.66	0.10	1.66	0.09	1.65	0.10	0.720	1.000	1.000	1.000
Body mass index (kg/m^2^)	23.55	4.55	23.56	5.83	23.19	4.67	0.908	1.000	1.000	1.000
**Lipid and apolipoprotein profile**										
Total cholesterol (mmol/L)	3.67	1.10	3.98	1.11	4.74*	2.28	0.003	0.054	1.000	0.003
Triglycerides (mmol/L)	0.95	0.54	1.49*	0.70	1.44*	0.65	< 0.001	1.000	< 0.001	0.001
High-density lipoprotein (mmol/L)	0.73	0.25	0.45*	0.22	0.50*	0.29	< 0.001	0.887	< 0.001	< 0.001
Low-density lipoprotein (mmol/L)	2.50	0.99	2.83	1.02	3.59*	2.33	0.003	0.054	0.922	0.002
Apolipoprotein C-III (µg/mL)	2665.76	2100.72	4519.96*	1808.15	4011.06*	1908.24	< 0.001	0.577	< 0.001	0.002
Apolipoprotein E (µg/mL)	220.90	79.60	340.68*^,^**	56.11	309.62*	53.04	< 0.001	0.049	< 0.001	< 0.001

TN, treatment-naïve participants; TE, treatment-experienced participants; *N*, sample size; HC, healthy control; mmHg, millimetres of mercury; vs, versus.

* , Values differ significantly between HIV-positive and HIV-negative (control group) (*p* < 0.05).

** , Values differ significantly between TN and TE (*p* < 0.05).

† , HIV-positive patients.

### Fasting lipid profile levels in patients and controls

All HIV-positive patients irrespective of ART experience had significantly higher mean values for total cholesterol (*p* = 0.003), triglycerides (*p* < 0.001), LDL-C (*p* = 0.003), ACIII (*p* < 0.001), and AE (*p* < 0.001) levels compared to the HIV-negative participants (Table 1). Conversely, the HDL-C concentrations was significantly lower (*p* < 0.001) among the HIV-negative patients compared to the HIV-positive patients. When the ART experience was considered, HIV-positive ART-experienced patients had significantly higher total cholesterol (*p* = 0.054), LDL-C (*p* = 0.054) and AE (*p* = 0.049) compared to the ART-naïve HIV-positive patients. No significant difference was noted when the triglycerides, HDL-C and ACIII values were compared among the ART-naïve and ART-experienced HIV-positive patients.

**TABLE 2:** Therapy consideration of the socio-clinical characteristics of the controls, antiretroviral therapy-naïve and treatment-experienced HIV-positive patients, Benin City, Nigeria, between May 2015 and November 2015.

Socio-clinical charateristics	Groups (mean ± s.d.)					*p*-value
	Healthy HIV-negative controls	Treatment-naïve HIV-positive participants	Treatment-experienced HIV-positive participants			
			< 1 year	1–2 years	> 2 years	
Age (years)	31.20 ± 7.26	39.36 ± 7.02	36.69 ± 7.51	37.67 ± 6.56	39.35 ± 8.03	0.567
Systolic blood pressure (mmHg)	130.72 ± 14.45	135.52 ± 24.79	141.31 ± 26.83	140.33 ± 33.82	144.65 ± 28.19	0.906
Diastolic blood pressure (mmHg)	85.50 ± 14.40	91.42 ± 21.85	93.77 ± 18.85	92.83 ± 26.99	92.06 ± 15.76	0.960
Weight (kg)	64.86 ± 12.11	65.10 ± 16.18	64.92 ± 9.60	58.33 ± 13.53	62.94 ± 13.12	0.562
Height (m)	1.66 ± 0.10	1.66 ± 0.09	1.69 ± 0.11	1.63 ± 0.09	1.64 ± 0.10	0.290
Body mass index (kg/m^2^)	23.55 ± 4.55	23.56 ± 5.83	22.95 ± 3.86	21.93 ± 4.55	23.53 ± 5.08	0.739

mmHg, millimetres of mercury.

### Therapy duration characteristics

There was no significant difference in the age, SBP, diastolic blood pressure, weight, height, and body mass index was of the various ART-duration groups (Table 2).

### Lipids and apolipoprotein levels based on therapy duration

There was a statistically significant difference when total cholesterol and LDL-C were compared among the various groups (Table 3). When the values of triglycerides, HDL-C, ACIII, and AE were compared for HIV-positive patients based on the duration of therapy, there were no statistically significant variations (Table 3).

**TABLE 3:** Therapy consideration of lipids and apolipoproteins for therapy duration of the controls, antiretroviral treatment-naïve and treatment-experienced HIV-positive patients, Benin City, Nigeria, between May 2015 and November 2015.

Lipids and apolipoprotein profile	Groups (mean ± s.d.)					*p*-value
	Healthy HIV-negative controls	Treatment-naïve HIV-positive participants	Treatment-experienced HIV-positive participants			
			< 1 year	1–2 years	> 2 years	
Total cholesterol (mmol/L)	3.67 ± 1.10	3.98 ± 1.11	3.95 ± 1.15	3.21 ± 1.05	5.37 ± 2.58	0.033
Triglycerides (mmol/L)	0.95 ± 0.54	1.49 ± 0.70	1.43 ± 0.46	1.28 ± 0.68	1.48 ± 0.72	0.799
High-density lipoprotein (mmol/L)	0.73 ± 0.25	0.45 ± 0.22	0.64 ± 0.37	0.52 ± 0.25	0.43 ± 0.24	0.088
Low-density lipoprotein (mmol/L)	2.50 ± 0.99	2.83 ± 1.02	2.66 ± 1.17	2.11 ± 0.84	4.26 ± 2.63	0.025
Apolipoprotein C-III (µg/mL)	2665.76 ± 2100.72	4519.96 ± 1808.15	4499.31 ± 1965.28	3055.33 ± 2019.60	3991.29 ± 1853.77	0.314
Apolipoprotein E (µg/mL)	220.90 ± 79.60	340.68 ± 56.11	329.77 ± 30.10	316.33 ± 50.14	299.87 ± 59.42	0.224

### Lipids and body mass index for patient staging

We observed no significant difference in the body mass index, total cholesterol, HDL-C, LDL-C, ACIII, and AE among both ART-experienced and ART-naïve HIV-positive patients in different stages of the disease. Stage 3 patients had significantly lower triglycerides levels (0.92 ± 0.27 mmol/L) compared with stage 4 patients (1.82 ± 0.78 mmol/L) but not significantly different compared to stages 1 and 2 patients (Table 4).

**TABLE 4:** Lipid and apolipoprotein levels among antiretroviral treatment-naïve and treatment-experienced HIV-positive participants per disease staging (WHO, 2005), Benin City, Nigeria, between May 2015 and November 2015.

Variables	Stage					*p*-value
	Stage 1 (*n* = 13)	Stage 2 (*n* = 12)	Stage 3 (*n* = 12)	Stage 4 (*n* = 13)	*F*-value	
**BMI (kg/m^2^) mean ± s.d.**						
Treatment-naïve	23.88 ± 4.45	26.57 ± 5.05	24.02 ± 6.44	21.51 ± 6.41	1.740	0.172
Treatment-experienced	23.89 ± 4.16	24.52 ± 5.46	21.58 ± 4.10	22.36 ± 4.97	1.216	0.315
**Total cholesterol (mmol/L) mean ± s.d.**						
Treatment-naïve	4.11 ± 1.31	3.73 ± 1.32	3.96 ± 1.21	4.03 ± 0.80	0.228	0.876
Treatment-experienced	5.02 ± 2.19	5.51 ± 2.91	4.12 ± 1.81	3.63 ± 0.77	1.486	0.231
**Triglycerides (mmol/L) mean ± s.d.**						
Treatment-naïve	1.40 ± 0.68	1.53 ± 0.53	0.92 ± 0.27†	1.82 ± 0.78	4.050	0.012
Treatment-experienced	1.21 ± 0.69	1.59 ± 0.76	1.56 ± 0.56	1.28 ± 0.29	1.109	0.355
**High-density lipoprotein (mmol/L) mean ± s.d.**						
Treatment-naïve	0.47 ± 0.21	0.48 ± 0.19	0.50 ± 0.31	0.38 ± 0.19	0.894	0.451
Treatment-experienced	0.56 ± 0.32	0.52 ± 0.25	0.41 ± 0.31	0.56 ± 0.28	0.840	0.479
**Low-density lipoprotein (mmol/L) mean ± s.d.**						
Treatment-naïve	2.96 ± 1.26	2.55 ± 1.19	3.04 ± 1.09	2.78 ± 0.71	0.443	0.724
Treatment-experienced	3.92 ± 2.47	4.27 ± 2.84	3.00 ± 1.84	2.48 ± 0.79	1.254	0.301
**Apolipoprotein C-III (µg/mL) mean ± s.d.**						
Treatment-naïve	4698.54 ± 2026.08	4942.00 ± 1353.02	4353.89 ± 1809.90	4239.56 ± 1946.34	0.378	0.769
Treatment-experienced	3199.79 ± 2143.42	4395.87 ± 1556.47	4184.94 ± 1942.34	4571.80 ± 1917.29	1.254	0.301
**Apolipoprotein E (µg/ml) mean ± s.d.**						
Treatment-naïve	325.08 ± 71.99	347.80 ± 45.42	338.89 ± 77.23	348.89 ± 35.04	0.504	0.681
Treatment-experienced	295.50 ± 80.50	306.20 ± 43.52	321.25 ± 34.08	322.20 ± 30.58	0.688	0.564

Note: *F*-values – analysis of variance of the studied biochemical parameters of the HIV-positive patients at different stages of the disease.

BMI, body mass index; s.d., standard deviation.

† , Values differ significantly from stage 1.

### Associations between apolipoproteins C-III and E and lipid profiles

The triglycerides of the healthy controls and ART-naïve groups correlated positively with AE and ACIII. Among the ART-experienced group, triglycerides correlated positively with ACIII and negatively with AE (Table 5). There was a negative correlation between the total cholesterol and AE in the healthy controls and the treatment-naïve patients, while a positive correlation was observed with the ART-experienced group. With ACIII, total cholesterol correlated positively among the healthy control and treatment-experienced group and negatively with the treatment-naïve group. Among the healthy controls, a positive correlation was observed between HDL-C and the studied apolipoproteins. In the HIV-positive groups, HDL-C correlated negatively with the apolipoproteins. The LDL-C correlated negatively with AE among healthy controls and treatment-naïve group and positively in treatment-experienced groups. Low-density lipoprotein cholesterol correlated positively with ACIII in all studied groups. In all the parameters in the various groups, the associations between the apolipoprotein and lipids were not statistically significantly (*p*-value > 0.05).

**TABLE 5:** Pearson bivariate correlation between apolipoproteins C-III and E and the lipid profile of the controls, antiretroviral therapy-naïve and treatment-experienced HIV-positive patients, Benin City, Nigeria, between May and November 2015.

Lipid profile parameters	Apolipoprotein	Groups					
		Healthy HIV-negative controls		Treatment-naïve HIV-positive participants		Treatment-experienced HIV-positive participants	
		*r*-value	*p*-value	*r*-value	*p*-value	*r*-value	*p*-value
Total cholesterol (mmol/L)	AE (µg/mL)	−0.093	0.520	−0.003	0.982	0.055	0.707
	ACIII (µg/mL)	0.135	0.351	−0.032	0.825	0.068	0.638
Triglycerides (mmol/L)	AE (µg/mL)	0.079	0.586	0.210	0.143	−0.021	0.887
	ACIII (µg/mL)	0.152	0.292	0.143	0.688	0.199	0.167
High-density lipoprotein (mmol/L)	AE (µg/mL)	0.021	0.883	−0.184	0.201	−0.138	0.340
	ACIII (µg/mL)	0.222	0.121	−0.140	0.331	−0.222	0.122
Low-density lipoprotein (mmol/L)	AE (µg/mL)	−0.128	0.376	−0.077	0.596	0.073	0.613
	ACIII (µg/mL)	0.055	0.704	0.108	0.454	0.069	0.634

Note: *r*-value = Pearson correlation coefficient.

ACIII, apolipoprotein C-III; AE, apolipoprotein E.

## Discussion

The present study assessed and compared the lipid profile, ACIII, and AE among HIV-positive ART-naïve patients and those on ART. We found that the studied apolipoproteins and lipid profiles of HIV-negative participants were often lower than those of HIV-positive participants irrespective of the treatment experience. This suggests that HIV itself disrupts apolipoprotein concentrations, consequently disrupting lipid metabolism.

We observed a significantly high SBP among the ART-experienced compared to the healthy control participants. Hypertension is known to increase the risk of cardiovascular disease and atherosclerosis. Previous studies conducted in Ethopia in 2018 and Uganda between 2011 to 2014 have reported hypertension to be highly prevalent among HIV patients on ART treatment.^23,24,25,26^ HIV patients on ART are highly susceptible to developing hypertension compared to HIV-negative persons. The pathophysiology of hypertension among HIV patients may be directly or indirectly influenced by the virus or treatment-related factors. Chronic inflammation and lipodystrophy associated with HIV infection have effects on the renin-angiotensin-aldosterone pathway, which may lead to kidney damage, vascular dysfunction, alterations in sympathetic nervous outflow, and hence hypertension.^26^ The typical risk factors of hypertension include genetic predisposition and lifestyle.^23^ The results from this study suggest that HIV increases the risk of isolated systolic hypertension, which increases a person’s chance of cardiovascular-related mortality.^27^ Metabolic changes may contribute to the risk of developing isolated systolic hypertension.^28^

The serum concentrations of ACIII and AE were significantly higher in both ART-experienced and treatment-naïve patients compared to the controls, similar to the findings of Bonnet et al.,^29^ who also reported a marked increase of ACIII among patients on intensive ART receiving care in France in 1999. Apolipoprotein C-III plays a major role in the metabolism of triglyceride-rich lipoproteins, such as chylomicrons and very low-density lipoproteins. Apolipoprotein C-III impairs lipolysis by inhibiting lipoprotein lipase and also prevents the binding of AE and apolipoprotein B to receptors on the liver, thereby impeding hepatic clearance of these triglyceride-rich lipoproteins. In addition, ACIII also favours hepatic assembly and secretion of very low-density lipoproteins.^30^ Hence, increased serum concentration of ACIII is associated with decreased clearance of triglyceride-rich lipoproteins.

Triglyceride levels were unsurprisingly higher in both ART-experienced and treatment-naïve HIV-positive participants compared to the control group due to increased ACIII concentration. Serum triglyceride concentration was not significantly different between ART-naïve and ART-experienced HIV-positive patients. Previous studies conducted in Nigeria in 2010 and in India in 2014 have reported hypertriglyceridaemia in HIV patients^31,32^ and increasing serum triglyceride concentration with ART-duration.^32,33,34^ Thus, hypertriglyceridaemia may be influenced by HIV itself or ART use. HIV causes inflammation with the subsequent release of cytokines and decreased hepatic clearance, which may result in hypertriglyceridaemia.^6^ However, we did not observe a difference in the triglyceride concentration when therapy duration was considered; this may be attributed to our low sample size, which limits statistical strength.

Although an increase in age increases the risk of dyslipidaemia,^35,36^ prolonged use of ART may be a stronger predictor of dyslipidaemia in HIV patients. Our results did not record any difference in the lipid status of patients with different age groups. A high prevalence of dyslipidaemia has been associated with the prolonged use of first-line ART.^6^ Dyslipidaemia observed among patients on antiretroviral drugs in this study may be attributed to the effects of ART on lipid metabolism, as there was significantly high total cholesterol (*p* < 0.033) and LDL-C (*p* < 0.025) among patients on ART for more than two years. An increased LDL-C strongly predicts cardiovascular risk.^30,31^

Apolipoprotein E is a multifunctional protein secreted primarily in the liver. It plays a significant role in the plasma clearance of lipoproteins.^37^ High plasma concentrations of AE are a reflection of high circulating LDL-C and triglycerides. It has been described as an early and specific indicator of cardiovascular disease.^38^ Apolipoprotein E serum concentration between HIV-positive treatment-experienced and treatment-naïve groups was statistically significantly higher when compared to the HIV-negative population. This is similar to previous studies carried out in a south-eastern state of Nigeria in 2021, which reported significantly higher AE in HIV-positive patients compared to HIV-negative persons.^39^ Similar to our finding, Ezeugwunne et al.^40^ in 2019 reported a reduction in serum concentration of AE as the length of ART treatment progressed among HIV-positive patients in Nnawi, Nigeria. The reason for reduced AE with increased length of treatment is unclear but may be related to the immune system.^41^

### Limitations

The small sample size used for the analysis limited the statistical strength of the study.

### Conclusion

From this study, dyslipidaemia is associated with HIV infection with or without ART, although the pattern of dyslipidaemia may vary. Prolonged use of ART is a significant risk of dyslipidaemia among HIV-positive patients. Patients with HIV, therefore, are at a high risk of developing cardiometabolic diseases; hence, routine monitoring of these patients’ lipid profiles will benefit their management.
